# FUBP1: a new protagonist in splicing regulation of the *DMD* gene

**DOI:** 10.1093/nar/gkv086

**Published:** 2015-02-06

**Authors:** Julie Miro, Abdelhamid Mahdi Laaref, Valérie Rofidal, Rosyne Lagrafeuille, Sonia Hem, Delphine Thorel, Déborah Méchin, Kamel Mamchaoui, Vincent Mouly, Mireille Claustres, Sylvie Tuffery-Giraud

**Affiliations:** 1 Université Montpellier, UFR de Médecine, Montpellier F-34000, France; 2 Inserm U827, Laboratoire de Génétique de Maladies Rares, F-34000 Montpellier, France; 3 UR1199 Laboratoire de Protéomique Fonctionnelle, INRA, 34060 Montpellier cedex, France; 4 CHU Montpellier, Hôpital Arnaud de Villeneuve, Laboratoire de Génétique Moléculaire, F-34000 Montpellier, France; 5 Institut de Myologie, UM76 Université Pierre et Marie Curie (UPMC), Paris, France; 6 INSERM U 974, Paris, France; 7 CNRS UMR 7215, Paris, France

## Abstract

We investigated the molecular mechanisms for in-frame skipping of *DMD* exon 39 caused by the nonsense c.5480T>A mutation in a patient with Becker muscular dystrophy. RNase-assisted pull down assay coupled with mass spectrometry revealed that the mutant RNA probe specifically recruits hnRNPA1, hnRNPA2/B1 and DAZAP1. Functional studies in a human myoblast cell line transfected with *DMD* minigenes confirmed the splicing inhibitory activity of hnRNPA1 and hnRNPA2/B1, and showed that DAZAP1, also known to activate splicing, acts negatively in the context of the mutated exon 39. Furthermore, we uncovered that recognition of endogenous *DMD* exon 39 in muscle cells is promoted by FUSE binding protein 1 (FUBP1), a multifunctional DNA- and RNA-binding protein whose role in splicing is largely unknown. By serial deletion and mutagenesis studies in minigenes, we delineated a functional intronic splicing enhancer (ISE) in intron 38. FUBP1 recruitment to the RNA sequence containing the ISE was established by RNA pull down and RNA EMSA, and further confirmed by RNA-ChIP on endogenous *DMD* pre-mRNA. This study provides new insights about the splicing regulation of *DMD* exon 39, highlighting the emerging role of FUBP1 in splicing and describing the first ISE for constitutive exon inclusion in the mature *DMD* transcript.

## INTRODUCTION

Disruption of normal splicing has a key role as a direct cause of disease, a modifier of disease severity or as a determinant of disease susceptibility and therapeutic responses (1–3). Splicing depends on a complex regulatory code that specifies how, where and when mRNAs are assembled from their precursors (4). This code consists of loosely defined consensus sequences that define the splice junctions and of a myriad of auxiliary *cis*-acting splicing regulatory elements (SREs). Based on their position and function, SREs can be classified as exonic splicing enhancers (ESEs) and silencers (ESSs), intronic splicing enhancers (ISEs) and silencers (ISSs). These elements are bound by RNA-binding proteins that have either positive (primarily SR proteins) (5) or negative (primarily hnRNP proteins) (6,7) effects on spliceosome assembly in their vicinity to maintain appropriate constitutive splicing or to regulate alternative splicing. The intricate network of interactions among the many RNA-binding proteins is still poorly understood and there are many exonic and intronic elements for which *trans*-acting mediators remain to be identified (2).

The lethal X-linked Duchenne muscular dystrophy (DMD; MIM#310200) is caused by loss-of-function mutations in the *DMD* gene that result in the absence of the muscle protein dystrophin. Conversely, the mutations that allow the production of reduced levels of normal or truncated and partially functional dystrophin in muscle are associated with Becker muscular dystrophy (BMD; MIM#300376), a milder allelic variant of DMD. BMD is mostly caused by large deletions in the *DMD* gene that maintain an open reading frame (8). Unexpectedly, nonsense mutations that are usually associated with a severe phenotype due to premature termination of protein translation, account for up to 24% of BMD point mutations (9). BMD nonsense mutations are preferentially distributed in a subset of contiguous *DMD* in-frame exons (from exon 23 to exon 42) that encode the central rod domain of dystrophin, which is largely dispensable. Several studies reported phenotype attenuation *via* partial exclusion of the exon harboring the nonsense mutation. In these cases the preservation of an open reading frame allows the production of a partially functional protein (10–14). Based on *in silico* predictions, many of these mutations are presumed to disrupt ESE motifs (9), but very few of them have been extensively investigated to uncover the mechanisms underlying the alternative splicing events.

Antisense-mediated exon skipping to by-pass protein-truncating mutations from the dystrophin pre-mRNA is a promising therapeutic approach for DMD patients (15), which is currently being tested in clinical trials. In this context, a better understanding of splicing regulation of the *DMD* gene is required. Here we investigated the molecular basis of splicing alteration caused by a nonsense mutation in exon 39 (c.5480T>A, p.Leu1417*) identified in a patient with BMD. Exon 39 is part of the set of in-frame exons prone to exon skipping when mutated. We were particularly interested in characterizing the regulatory *cis*-elements and cellular *trans*-acting factors involved in this mutation-induced alternative splicing event. By using biochemical and functional approaches, we have established that the mutation affects RNA-protein complex formation by creating a splicing factor-dependent silencer. We also report that normal *DMD* exon 39 splicing in muscle cells is dependent on FUPB1, a largely unknown splicing factor.

## MATERIALS AND METHODS

### Dystrophin transcripts analysis

The muscular dystrophin mRNA analysis was performed in the patient in the course of the diagnostic procedure at the Hospital Laboratory. All tests have been approved by the local ethical committee and informed consent obtained. Amplification of dystrophin transcripts was carried out as described before (16) using primers located in exon 38 (forward) and in exon 40 (reverse) to visualize exon 39 skipping in presence of the c.5480T>A mutation. Mutation numbering is based on the *DMD* cDNA sequence (NM_004006.2), with the A of the translation start codon considered as nucleotide number 1.

### Splicing reporter minigenes and expression plasmids

*DMD* exon 39 and its flanking intronic sequences (272 nucleotides upstream and 262 nucleotides downstream of exon 39) were polymerase chain reaction (PCR)-amplified from genomic DNA isolated from a control individual and from the patient harboring the c.5480T>A mutation using High Fidelity Phusion Polymerase (Finnzymes). Amplicons were cloned in the XhoI and NheI sites of the pSPL3 vector (provided by I. Botillo, Department of Molecular Medicine, Sapienza University, Roma, Italy) to obtain the E39-WT and the E39-5480A splicing reporter minigenes, respectively. The same procedure was followed to generate the truncated (Δ1 and Δ2) minigenes. The Δ3 minigene was constructed by overlap extension PCR. Exon 39 minigenes carrying the deletion of the site O (del-O), the two U>A mutations in site O (O-mut) and/or the mutation of the UGU (UGU-mut) were obtained by mutagenesis of the E39-WT construct using the QuikChange II site directed-mutagenesis kit (Agilent Technologies). To generate expression plasmids, the FUBP1 (NM_003902.3), DAZAP1 (NM_170711.1) and hnRNPA1 (NM_031157.2) sequences were PCR-amplified with specific Flag-tagged primer pairs by using cDNA from HeLa cells. The obtained amplicons were cloned in the HindIII and XbaI (FBP and DAZAP1) or the EcoRI and XhoI (hnRNPA1) sites of pcDNA3.1+ (Life Technologies) and then sequenced. The pFlag CMV-4 expression vector for hnRNPA2 was provided by M. Baralle (ICGEB, Trieste, Italy). The list of the primers used in this study is available upon request.

### Cell culture, transfection and siRNA experiments

The human immortalized myoblast cell line C25cl48 established from a muscle biopsy of a healthy individual was used for all transfection experiments (17). Cells were grown at 37°C and 5% CO_2_ in Dulbecco's modified Eagle's medium (DMEM) with GlutaMAX and 199 medium (4:1 ratio) (Life Technologies) supplemented with 20% calf serum (Eurobio), 5 ng/mL hEGF, 0.5 ng/ml bFGF, 50 μg/ml bovine fetuin (Life Technologies), 5 μg/ml Insulin and 0.2 μg/ml dexamethasone (Sigma–Aldrich). C25cl48 myoblasts were plated in 6-well plates (1.8 × 10^5^ cells/well) and 1 μg of each splicing reporter minigene was transfected the following day using JetPEI^®^ (Polyplus Transfection) according to the manufacturer's instructions. For overexpression studies, the minigene was co-transfected with 100 ng of plasmid coding for the splicing factors or empty vector (negative control). Cells were harvested 48 h after transfection for RNA isolation or protein extraction.

For siRNA experiments in C25cl48 cells, two consecutive rounds of transfection were performed. In the first round of transfection, 2.5 × 10^5^ cells/well in six-well/plates were reverse transfected with 35 nM siRNA (Thermo Scientific Dharmacon) using 7.5 μl Lipofectamine^®^ RNAiMax (Life technologies) following the manufacturer's instructions. After 48 h, a forward transfection was performed using the same amounts of siRNA and reagent. Six hours after the second round of siRNA transfection, C25cl48 cells were transfected with 2.5 μg of minigene using Lipofectamine^®^ LTX (Life Technologies). Cells were harvested 48 h after minigene transfection. Previously described siRNAs were used for silencing *hnRNPA1, hnRNPA2B1, DAZAP1* (18) and *FUBP1* (#1 in (19)). The SiGENOME non-targeting siRNA#1 was used as negative control.

### RT-PCR assays

Total RNA was extracted using the RNeasy Plus Mini Kit (Qiagen). Superscript^®^ II (Life Technologies) and random primers were used to reverse transcribe 900 ng of total RNA. An aliquot (1/20th) was then PCR-amplified with the pSPL3 specific primers as previously described (20). Heteroduplexes were eliminated from PCR products by reconditioning PCR for three cycles (21). After electrophoresis on agarose gels, the Quantity One (v.4.6.9) software (Bio-Rad) was used to quantify the spliced products. Percentage of *DMD* exon 39 skipping was calculated as the ratio of the intensity of the lower band (excluding exon 39) to the sum of the intensities of the upper and lower bands.

### Western blotting

Proteins were separated on 10% sodium dodecyl sulphate (SDS)-polyacrylamide gels, transferred to Polyvinylidene fluoride (PVDF) membranes and probed using specific antibodies: polyclonal anti-DAZAP1 (kindly provided by F. Pagani, ICGEB, Trieste, Italy), anti-hnRNPA1 (4B10), anti-hnRNPA2B1 (DP3B3) (Santa Cruz Biotechnologies) and anti-FUBP1 (Sigma–Aldrich). Anti-ß-Tubulin (AA2, Millipore) was used as loading control.

### *In vitro* transcription, standard and RNase-assisted RNA pull down

The annealed sense and antisense oligonucleotides (MWG) corresponding to the different RNA probes were cloned into the EcoRI–PstI-digested pGEM^®^-3Z vector (Promega). HindIII linearized templates were then *in vitro* transcribed using RiboMAX™ Large Scale RNA Production System-T7 (Promega). The control non-specific GST-80 RNA probe was previously described (22). RNA probes (700 pmol) were oxidized with 5 mM sodium *m*-periodate in 100 mM sodium acetate, pH 5.0 (Sigma–Aldrich) on a rotator in the dark for 1 h. RNA probes were purified with the RNeasy Plus Mini Kit (Qiagen) using the ‘Purification of total RNA containing miRNA’ Qiagen supplementary protocol to retain small RNAs. The standard RNA pull down experiments were performed as previously described (23) with some modifications. First, each oxidized RNA probe was bound to 250 μl adipic acid dihydrazide agarose beads (Sigma–Aldrich). Then, the immobilized RNA was incubated with 80 μl HeLa cell nuclear extract (i.e. 0.5 mg proteins) (Promega) in 70 μl buffer D, supplemented with 3.2 mM MgCl_2_, 67 μg *Escherichia coli* tRNA (Sigma–Aldrich) and 12 μg bovine serum albumin (NEB), at 30°C for 30 min. The beads were then pelleted by centrifugation at 100 g for 2 min and washed four times with buffer D containing 1.5 mM MgCl_2_ before addition of Laemmli buffer and loading on a 10% SDS-PAGE gel. Pulled-down proteins were analyzed by western blotting using specific antibodies. The RNase-assisted RNA pull down assay was conducted using the same procedure except that 1500 pmol of RNA probes were used. After the fourth wash with 1.5 mM MgCl_2_–buffer D, beads were additionally washed twice with Diethylpyrocarbonate-treated-water and proteins specifically bound to RNAs were eluted by using a RNase A/T1 treatment as previously described (24). RNA-bound proteins were separated by SDS-PAGE and silver-stained (PlusOne Silver Staining Kit, GE Healthcare). Selected bands were excised and proteins identified by mass spectrometry analysis (detailed procedure in Supplementary Method).

### RNA EMSA

*In vitro* transcribed RNAs were biotinylated using the Pierce RNA 3′ End Biotinylation Kit (Thermo Scientific) according to the manufacturer's instructions. The gel shift assay was carried out using the LightShift Chemiluminescent RNA EMSA Kit (Thermo Scientific). Biotinylated wild-type or mutant RNA probe (20 fmol) were incubated 30 min at room temperature with recombinant FUBP1 (Origene) in a 20 μl binding reaction containing 1× binding buffer, 5% glycerol and 0.1 mg/ml tRNA. The samples were electrophoresed on a 5% native PAGE in 0.5× Tris Borate EDTA, transferred to a positively charged nylon membrane (Pall) and crosslinked in a UV Stratalinker 1800 (Stratagene). Detection of biotin-labeled RNA probes was performed using the chemiluminescent nucleic acid detection module (Thermo Scientific).

### RNA-ChIP

RNA chromatin immunoprecipitation (RNA-ChIP) was performed using RNA ChIP-IT^®^ (Active Motif) according to the manufacturers’ instructions. Briefly, C25cl48 cells were cross-linked for 5 min with 1% formaldehyde. Chromatin was sheared using the Diagenode Bioruptor (3 cycles of 30 s ‘on’ and 30 s ‘off’ in ice-cooled water). Immunoprecipitation was performed with 14 μg chromatin and 4 μg polyclonal anti-FUBP1 antibody (GeneTex), or normal rabbit IgG (Santa Cruz Biotechnology). After purification, RNA was treated with DNase I. Superscript III reverse transcriptase (Life Technologies) and random hexamers were used to convert one-third of the recovered RNA. Products were amplified for 35 cycles from cDNA using the following primers forward 5′-AAGGCTATGAGCACAGTATC-3′ and reverse 5′- AAAGCTGTACATTGTTAACAGAG-3′.

### Microarray analysis

Total RNA samples from human skeletal muscle (purchased from Ambion, AM7982) and from C25cl48 cells were processed, hybridized on a GeneChip^®^ Human Gene 2.0 ST Array, scanned, and quantified at the Affymetrix Service Provider and Core Facility, ‘KFB – Center of Excellence for Fluorescent Bioanalytics’ (University of Regensburg, Regensburg, Germany; www.kfb-regensburg.de). A heat-map image of microarray data was generated from log_2_ values using the Matrix2png web interface (University of British Columbia, http://chibi.ubc.ca/matrix2png; (25)).

## RESULTS

### The c.5480T>A mutation causes *DMD* exon 39 skipping

**Figure 1.**
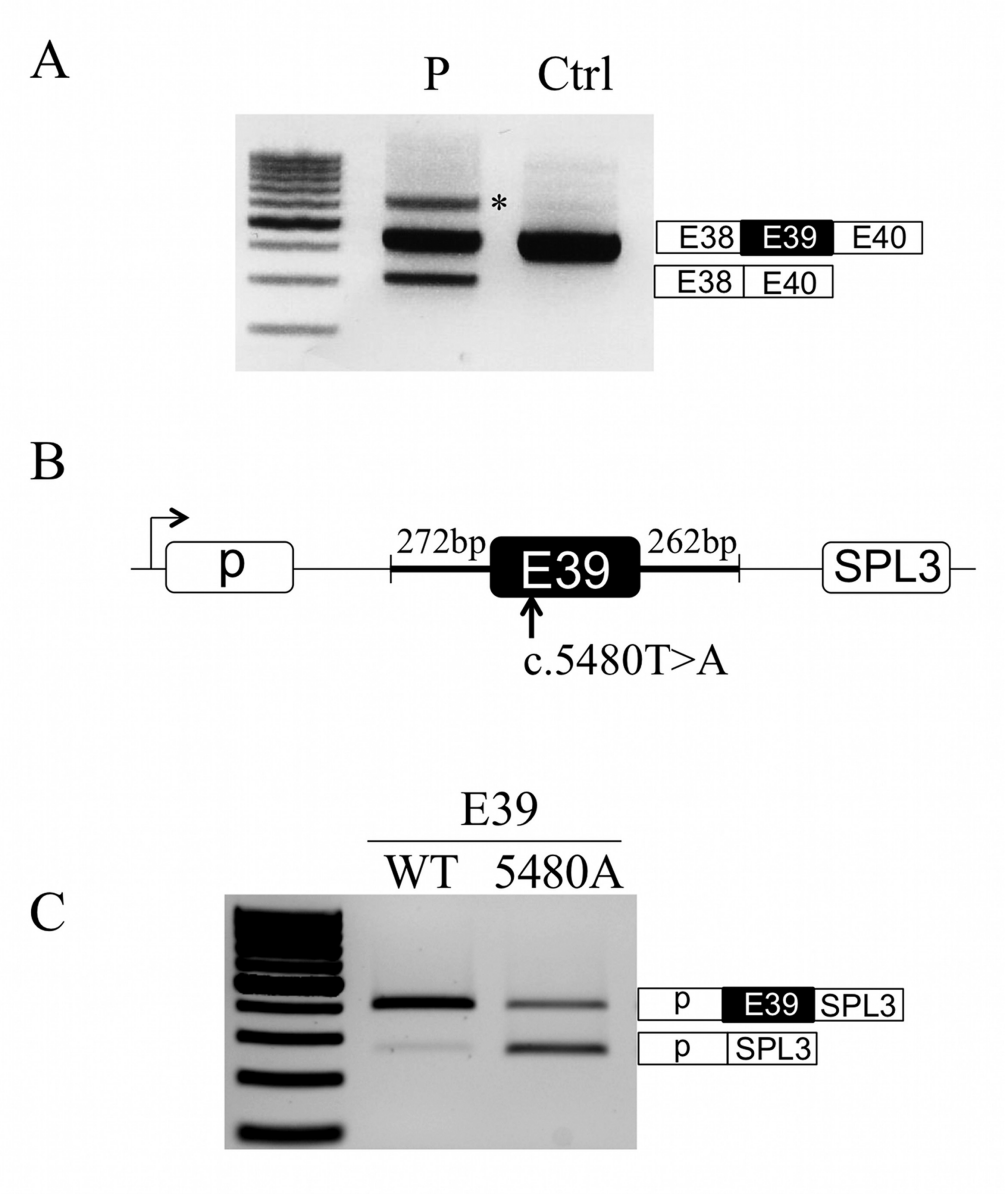
The c.5480T>A mutation in the *DMD* gene causes partial exon 39 skipping. (**A**) Dystrophin transcripts analysis. Muscle dystrophin transcripts were amplified by RT-PCR in the patient harboring the c.5480T>A mutation (P) and a control (Ctrl) using primers located in exons 38 (forward) and 40 (reverse). The identity of each transcript was verified by Sanger sequencing and was indicated at the right side of the gel. The asterisk (*) shows the position of heteroduplex PCR products. (**B**) Schematic representation of the pSPL3 splicing reporter minigene. *DMD* exon 39 (dark box) and flanking intronic sequences (thick lines) amplified from a normal control or the patient carrying the c.5480T>A mutation were cloned in the pSPL3 vector (thin lines) between its two exons (white boxes). (**C**) Minigene splicing assays. C25cl48 human myoblasts were transiently transfected with the wild-type (E39-WT) or mutated (E39-5480A) pSPL3 minigene. The splicing pattern was analyzed by RT-PCR using minigene-specific primers. The identity of each transcript was verified by Sanger sequencing and was indicated at the right side of the gel.

The nonsense c.5480T>A (p.Leu1827*) mutation in *DMD* exon 39 was identified in a 27-year-old patient having a Becker-like phenotype. The milder than expected phenotype arises because of partial skipping of exon 39 in muscle, which removes the nonsense mutation and reframes the dystrophin transcripts (Figure 1A). To recapitulate in a heterologous system the splicing pattern observed in the patient, we made minigene constructs in which the sequence of either *DMD* exon 39 wild-type (WT) or harboring the c.5480T>A was cloned in the pSPL3 splicing reporter vector (Figure 1B). The two minigenes (E39-WT, E39-5480A) were transiently transfected in the C25cl48 human myoblast cell line (17) and the splicing pattern was assessed by reverse transcriptase-polymerase chain reaction (RT-PCR) analysis (Figure 1C). The exon 39 was almost totally included in WT condition while skipping occurred in the minigene harboring the c.5480T>A mutation, reproducing the splicing defect observed in the patient.

### The silencer gain-of-function model

**Figure 2.**
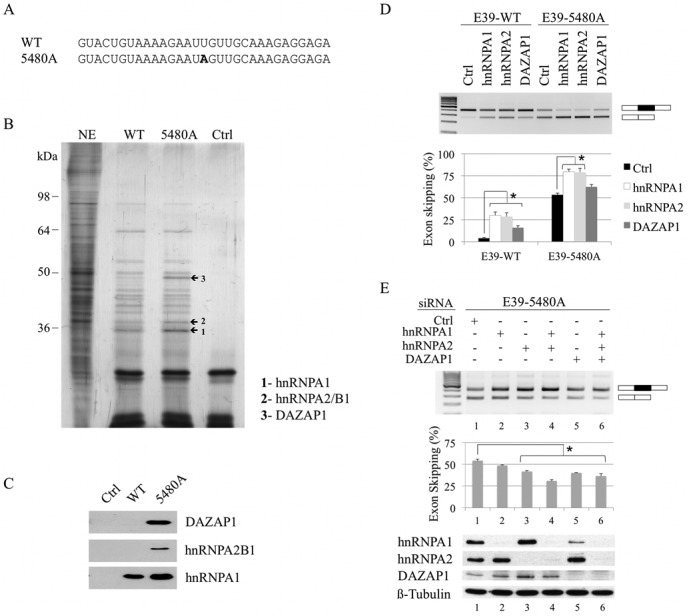
The splicing factors hnRNPA1, hnRNPA2B1 and DAZAP1 are preferentially recruited to the 5480A-mutated probe and promote exon 39 skipping. (**A**) Sequences of the WT and the mutated (5480A) RNA probes used for the RNA pull down experiments. The position of the 5480A mutation is in bold. (**B**) Study of hnRNPA1, hnRNPA2B1, and DAZAP1 binding by RNAse-assisted RNA pull down assay. The RNA probes were bound to adipic acid dihydrazide agarose beads and incubated with HeLa cell nuclear extract (NE). Proteins specifically bound to the RNA probes were eluted from beads by RNase treatment, separated on 10% SDS-PAGE gels and silver-stained. The proteins from bands 1 to 3 (arrows) were identified by mass spectrometry as hnRNPA1 (1), hnRNPA2B1 (2) and DAZAP1 (3). NE, nuclear extract; Ctrl, experiment performed without RNA probe. (**C**) Western blotting analysis of standard RNA pull down assays. The RNA probes bound to the adipic acid dihydrazide agarose beads were incubated with HeLa cell nuclear extract. After washing, pulled-down proteins were specifically detected by western blotting using antibodies directed against DAZAP1, hnRNPA2B1 and hnRNPA1. Ctrl, experiment performed without RNA probes. The experiment was repeated twice independently. (**D**) Effect of hnRNPA1, hnRNPA2 and DAZAP1 overexpression on exon 39 splicing. C25cl48 myoblasts were co-transfected with the E39-WT or E39-5480A minigene and hnRNPA1, hnRNPA2 and DAZAP1 expressing plasmids, or empty pcDNA3.1+ vector (control, Ctrl). Upper panel: RT-PCR analysis of exon 39 splicing pattern. The resulting products are indicated at the right side of the gel with alternatively spliced exon 39 (black box) and flanking vector exons (white boxes). Lower panel: quantification of exon 39 skipping percentage (mean ± SEM of four independent transfections). The result of the Wilcoxon rank-sum test is given (*P < 0.05) (**E**) Effect of hnRNPA1, hnRNPA2 and DAZAP1 depletion on mutant exon 39 splicing. After transfection with the siRNAs targeting *hnRNPA1, hnRNPA2* or *DAZAP1*, C25cl48 myoblasts were transfected with the E39-5480A minigene. A non-targeting siRNA was used as negative control (Ctrl). Upper and middle panels: RT-PCR analysis, exon skipping quantification, and statistical analysis were as in Figure 2D. Lower panel: siRNA efficiency assessed by western blotting.

We next investigated the molecular basis of the splicing defect induced by the c.5480T>A mutation. We used an RNase-assisted RNA pull down assay to compare the *trans*-acting factors that differently bind to the wild-type (WT) and the mutated (5480A) RNA probes (Figure 2A). This modified pull down method allows a more sensitive and thorough detection of RNA-binding proteins that recognize a specific RNA sequence (24). Comparison of the binding patterns revealed that three protein bands (bands 1–3) were more intense in the lane corresponding to the 5480A RNA probe (Figure 2B). Mass spectrometry analysis indicated that these bands correspond to hnRNPA1, hnRNPA2/B1 and DAZAP1 proteins. These results were independently confirmed by standard RNA pull down assays followed by immunoblotting with anti-DAZAP1, -hnRNPA2/B1 and -hnRNPA1 antibodies (Figure 2C).

To address the functional role of hnRNPA1, hnRNPA2 and DAZAP1 in the recognition of mutated *DMD* exon 39, vectors encoding each of the three proteins were co-transfected with the E39-WT or E39-5480A minigene in C25cl48 cells. The overexpressed Flag-tagged proteins were visualized by western blotting using an anti-Flag antibody (Supplementary Figure S1). RT-PCR analysis of the transcripts derived from the E39-5480A minigene showed that overexpression of all three proteins, but especially hnRNPA1 and hnRNPA2 proteins, led to a marked increase in exon 39 skipping (82% for hnRNPA1 and 79% for hnRNPA2) as compared to the control (53%) (Figure 2D). Overexpression of hnRNPA1, hnRNPA2 and DAZAP1 also enhanced the proportion of skipped exon 39 in the E39-WT minigene, suggesting the presence of binding sites for these inhibitory proteins in the wild-type sequence. Then, we used specific siRNAs to silence *hnRNPA1, hnRNPA2* and *DAZAP1* in C25cl48 cells that were transfected with the E39-5480A minigene. All siRNAs significantly reduced the level of target protein (Figure 2E, bottom). As previously reported (26), hnRNPA1 expression was also markedly decreased in cells transfected with the siRNA targeting *DAZAP1* (lane 5), whereas hnRNPA2 was not affected. SiRNA-mediated depletion of each individual protein resulted in a significant decrease in mutated exon 39 skipping only in hnRNPA2-, and DAZAP1-silenced cells (42% and 39% exon skipping, respectively) compared to control siRNA (54% exon skipping). Double (*hnRNPA1/hnRNPA2*) or triple knockdown (*hnRNPA1/hnRNPA2/DAZAP1*) further reduced the percentage of exon 39 skipping (29.5% and 34%, respectively). Altogether, these data provide strong support to the hypothesis that the c.5480T>A mutation leads to the creation of a hnRNPA1/A2-dependent ESS that plays a significant role in exon 39 skipping and that DAZAP1 is also associated with this silencer complex.

### Differential binding of FUBP1 in RNA pull down experiments

In order to normalize the amount of pulled-down RNAs, the RNase-assisted RNA pull down experiment was also conducted with modified RNA probes containing a TDP43 binding (ug)6-repeat linked by an u5 spacer as previously described (26). The identity of bands 1–3 was reconfirmed in this experiment (Supplementary Figure S2A and S2B), but a differential binding to the WT and mutated probes was also detected for a protein of about 65 kDa (band 4). The mass spectrometry analysis indicated that the band 4 corresponds to Far upstream element (FUSE)-binding protein 1 (FUBP1), a largely nuclear protein with both DNA- and RNA-binding activity (27). Here, contrary to the binding pattern observed for bands 1 to 3, the protein band was enriched in the wild-type (ug)6u5-WT RNA probe lane compared to the (ug)6u5-5480A mutated RNA probe lane, suggesting that the affinity of FUBP1 decreases when the mutation is present. However, FUBP1 was found not to bind to either the WT or to the mutated exon 39 RNA probe lacking the (ug)6u5-extension (Figure 2B). A standard RNA pull down assay followed by immunoblotting with specific anti-FUBP1 and -TDP43 (loading control) antibodies confirmed the differential binding of the protein depending on the probes (Supplementary Figure S2C). Literature data about FUBP1 binding properties provided some clues to explain this result. While FUBP1 was described to bind to AU-rich elements (AREs) in 3′-UTR of several pre-mRNAs (27), in this study, the FUBP1 binding sequence is more similar to the TG-rich ssDNA binding motif previously identified by SELEX. The reported optimal binding sequences for the hnRNP K homology (KH) domains 2 to 4 are T(T/C)GT, and (T/G)TG(T/C) for the KH1 domain (28,29) (Supplementary Figure S3). Inspection of the wild-type exon 39 sequence revealed that the 5480 position lies within a UUGUUGC sequence composed of two potential overlapping FUBP1 binding sites UUGU and UUGC, but no binding was evidenced in the RNA pull down experiments (Supplementary Figure S2C). By contrast FUBP1 was recruited when the extra (ug)6u5-tail was added ((ug)6u5-WT). This was attributed to the presence of six additional FUBP1 potential binding sites (five overlapping GUGUs and one UUGU). Interestingly, the disruption of the UUGUUGC sequence (UUGUUGC > UAGUUGC) at the 3′ end of the probe and outside the (ug)6u5-tail ((ug)6u5-5480A) strongly reduced the binding of FUBP1, showing that the binding of FUBP1 to the UG-rich probe is reinforced by the presence of the distal sequence.

### FUBP1, a positive acting factor for *DMD* exon 39 splicing

**Figure 3.**
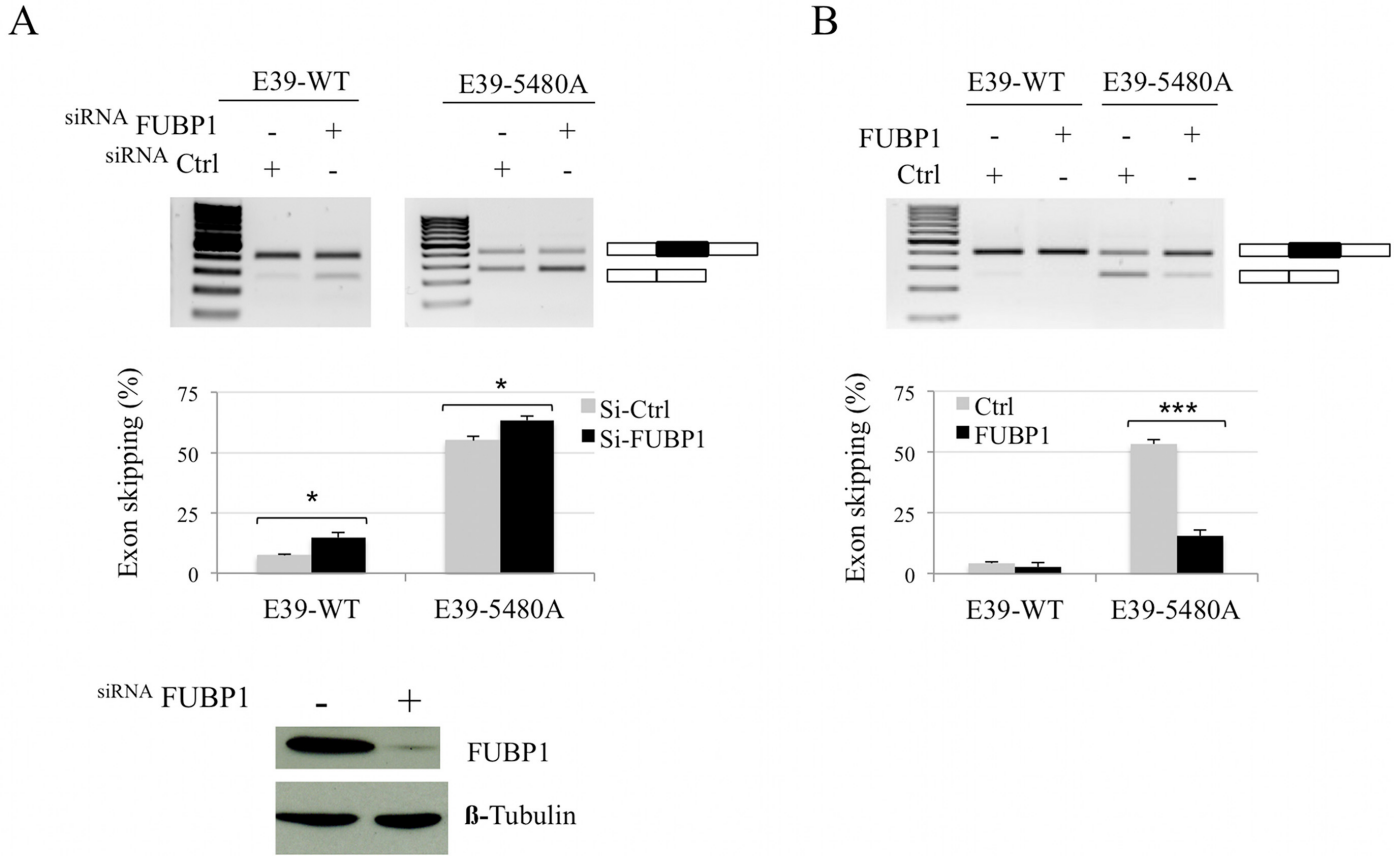
FUBP1 promotes *DMD* exon 39 inclusion. (**A**) *FUBP1* depletion induces *DMD* exon 39 skipping in the minigene. C25cl48 myoblasts were transfected with siRNAs targeting either *FUBP1* or *firefly luciferase* (siCtrl) and the E39-WT or E39-5480A minigene. Upper and middle panels: RT-PCR analysis of exon 39 splicing pattern using vector-specific primers. RT-PCR analysis, exon skipping quantification, and statistical analysis were as in Figure 2D. Lower panel: siRNA treatment efficiency assessed by western blotting. (**B**) FUBP1 overexpression leads to inclusion of exon 39 in the minigene. E39-WT or E39-5480A minigene were co-transfected into C25cl48 myoblasts along with the FUBP1 expressing plasmid or empty pcDNA3.1+ vector (Ctrl). RT-PCR analysis and exon skipping quantification were as in (A) except that the experiments were repeated four and seven times independently with the E39-WT and the E39-5480A minigenes, respectively (***P < 0.001).

We sought to clarify whether FUBP1 may have a functional role on *DMD* exon 39 splicing. To this aim, C25cl48 myoblasts treated with either siRNAs targeting *FUBP1* or *firefly luciferase* as a control were transfected with the E39-WT or E39-5480A minigene (Figure 3A). Depletion of FUBP1 induced a statistically significant increase in exon 39 skipping, and in the same range in the wild-type and mutated minigenes, indicating that FUBP1 acts as a splicing transactivator of *DMD* exon 39 independently of the 5480T>A mutation. To confirm the activating role of FUBP1 on exon 39 splicing, we generated a cDNA construct coding for FUBP1 and analyzed the splicing pattern of the E39-WT and E39-5480A minigene under overexpression of FUBP1 in C25cl48 cells (Figure 3B). As expected, FUBP1 overexpression had no effect on inclusion of WT exon 39 since it was already nearly 100% included. By contrast, in the 5480A-mutated minigene context initially harboring 53% exon 39 skipping, FUBP1 overexpression reduced exon skipping drastically to levels as low as 15%, thus confirming that FUBP1 is a strong splicing activator of *DMD* exon 39. Our results indicate that positive splicing regulation of *DMD* exon 39 by FUBP1 is not dependent on the sequence around position 5480. This finding correlates well with the absence of binding observed in the pull down experiments when the exon 39-specific RNA probes were used. This hypothesis is further reinforced by the absence of detectable exon 39 skipping in transfection experiments with E39-WT minigene deleted for the two predicted binding sites around position 5480 (Supplementary Figure S4).

### An FUBP1 binding site in intron 38 is crucial for recognition of *DMD* exon 39

**Figure 4.**
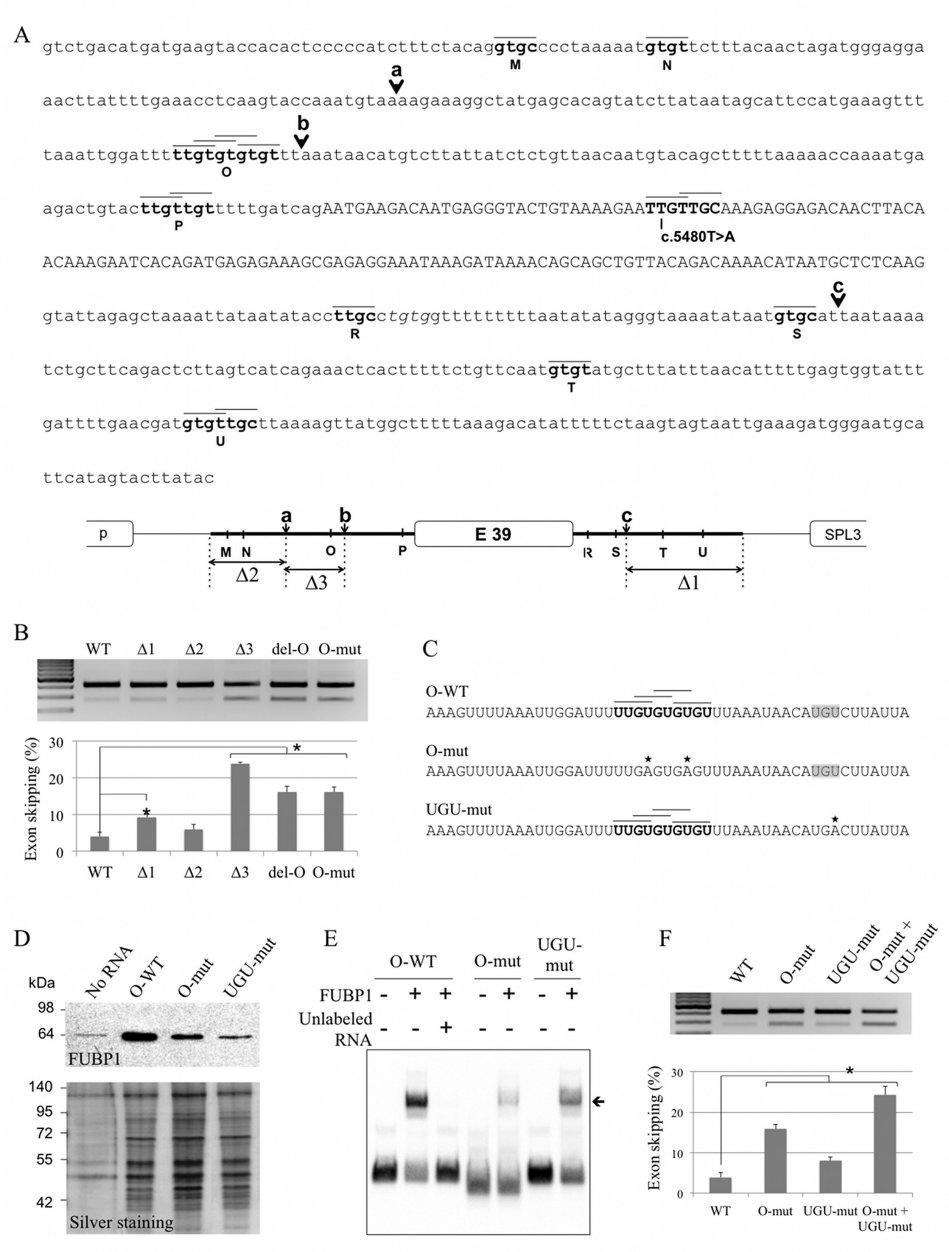
Identification of a functional FUBP1 binding site in *DMD* intron 38. (**A**) Predicted potential FUBP1 binding sites in exon 39 and its flanking intronic sequences. Upper panel: sequence of the *DMD* exon 39 (in upper case) and its flanking intronic sequences, including rs34281911, cloned in the WT minigene. The potential FUBP1 binding sites (M, N, O, P, R, S, T, U, in bold) were determined using SELEX-identified ssDNA binding motifs, T(T/C)GT and (T/G)TG(T/C) for FUBP1 KH2 to 4 and for FUBP1 KH1, respectively (29). Black lines above the sequence represent each tetrad matching the binding motif. Black arrows indicate the truncation sites (a, b, c) in the minigenes used in the serial deletion analysis. Lower panel: schematic representation of the internal deletions (Δ1, Δ2, Δ3) in the E39-WT minigene. The white boxes represent exons, the thin black line is for pspl3 intronic sequence and the thick black line is for *DMD* intronic sequence. (**B**) The site O is an Intronic Splicing Enhancer. RT-PCR analysis of transcripts derived from a series of E39 minigenes transfected in C25cl48 myoblasts: WT, truncated (Δ1, Δ2 or Δ3), deleted (del-O, del-UUGUGUGUGU) or mutated (O-mut) in site O (as described in 4C). RT-PCR analysis, exon skipping quantification, and statistical analysis were as in Figure 2D. (**C**) Nucleotide sequence of the RNA probes designed for the binding experiments. The wild-type RNA probe (O-WT) contains the four overlapping predicted FUBP1 binding sites (black horizontal lines above the sequence) of the site O (in bold). In the mutated probes (O-mut, UGU-mut), the mutated nucleotides are marked with an asterisk. The two U>A mutations in the O-mut probe disrupt the four predicted binding tetrads. The U>A mutation in the UGU-mut probe disrupts an UGU motif (highlighted in gray). (**D**) FUBP1 binds to the ISE-containing site O in *DMD* intron 38. The standard RNA pull down assay was performed as in Figure 2C. Upper panel: western blot analysis of the pull down assay using an anti-FUBP1 antibody. Lower panel: silver-staining of the pulled down proteins separated by SDS-PAGE to visualize the amount of proteins loaded. (**E**) FUBP1 directly binds to the wild-type RNA probe that contains site O. The O-WT or mutated (O-mut, UGU-mut) biotinylated RNA probes (20 fmol) were incubated 30 min at room temperature with 200 nM of recombinant FUBP1 protein and resolved on a 5% native polyacrylamide gel. The arrow indicates band-shifted complexes. For the competition assay, an excess of unlabeled O-WT RNA probe (200 pmol) was added. (**F**) The UGU element in close vicinity of the O site contributes to the functional ISE. C25cl48 myoblasts were transfected with the WT minigene or minigenes carrying the O-mut or the UGU-mut mutation alone (as described in (C)) or in combination (O-mut+UGU-mut). RT-PCR analysis, exon skipping quantification, and statistical analysis were as in Figure 2D.

Next, we searched for other potential FUBP1 binding sites in the *DMD* genomic sequence cloned in the minigenes. We looked for tetrads perfectly matching with the previously described ssDNA FUBP1 binding motif (Supplementary Figure S3) (29). Besides the exonic cluster around the c.5480 position, eight potential intronic sites (M, N, O, P, R, S, T, U) were identified, of which one (site O) consisted of four overlapping FUBP1 putative binding sites (Figure 4A). To assess the role of the predicted FUBP1 binding sequences on exon 39 splicing, we transfected C25cl48 cells with serially deleted E39-WT minigenes (Δ1, Δ2, Δ3) and the splicing pattern was analyzed by RT-PCR as before (Figure 4A and B). The Δ3 deletion caused a strong increase in exon 39 skipping (24%) while the two Δ1 and Δ2 deletions had little (9%, Δ1) or no (6%, Δ2) impact on exon 39 splicing compared to the wild-type (4%). Even though the presence of other regulatory elements in the surrounding sequence can not be ruled out, the high level of exon skipping induced by the Δ3 deletion led us to focus on this intronic region. We assumed that a regulatory element essential for proper exon 39 inclusion in the mature transcripts was present within this sequence, possibly the predicted FUBP1 binding site O. Consistently, deletion or mutagenesis of site O in the E39-WT minigene construct (del-O, del UUGUGUGUGU; O-mut, two T>A mutations as described in Figure 4C) increased exon 39 skipping (16% for each construct compared to 4% with the E39-WT minigene). The effect of the O-mut mutation on exon skipping was also observed in the E39-5480A minigene context (70% for the O-mut+5480A versus 53% for the 5480A, Supplementary Figure S5). These observations indicate that the site O could be an ISE.

Then, we assessed the ability of FUBP1 to bind to the identified intronic RNA motif by standard RNA pull down assays. As shown by Western blot analysis, the wild-type site O-containing probe was strongly bound by FUBP1, and disruption of the four predicted binding motifs in the double mutant probe (O-mut) was shown to decrease this interaction (Figure 4C and D). We further investigated the role of the TGT sequences previously identified as the core of the ideal binding site by ssDNA SELEX and presenting a strong interference with FUBP1 in the FUSE region (Supplementary Figure S3, (29)). To this end, we mutated the UGU sequence located 11 bp downstream of the site O (UGU-mut) by one single U>A mutation. Indeed we observed that the mutation also strongly reduced the binding of FUBP1 to the RNA probe in the RNA pull down experiments (Figure 4D).

We next carried out RNA EMSA experiments using 3′-end biotinylated wild-type and mutated RNA probes to confirm direct RNA–protein interaction of FUBP1 (Figure 4E). The recombinant FUBP1 protein formed complexes with the O-WT RNA probe in a concentration dependent manner (Supplementary Figure S6). The concentration of FUBP1 was then set to 200 nM for the following experiments. While the O-WT probe was predominantly bound to FUBP1, both mutant probes (O-mut and UGU-mut) were much less efficiently shifted confirming the importance of these two sequence motifs for optimal binding of FUBP1. The binding specificity was checked by displacement of the bound fraction with an excess of unlabeled RNA probe (Figure 4E, lane 3).

Overall, the RNA pull down and the RNA EMSA experiments consistently show on the one hand the ability of FUBP1 to bind to the wild-type RNA sequence efficiently, and on the other hand a marked decrease in binding to RNA probes mutated in the site O or in the sequence UGU. Depending on the technique, we noticed that this decrease was stronger either for the O-mut or the UGU-mut probe. We assumed that these variations can be due to the technical differences between the two methods.

We finally investigated the functionality of the UGU sequence. Therefore, we analyzed the splicing pattern of the minigene with the mutated UGU sequence (U>A mutation as described in Figure 4C) alone or in combination with the mutation at site O (Figure 4F). After transfection of the UGU-mut minigene in C25cl48 myoblasts, a sligth but significant increase in exon 39 skipping was observed (8% compared to 4% for the WT construct). These results are fully in line with the data obtained from RNA–protein interaction experiments. Likewise, the mutagenesis of the UGU motif in the O-mut construct led to 24% exon skipping (compared to 16% for the O-mut construct alone), supporting an additive effect of the O and the UGU elements composing the ISE.

### FUBP1 is required for the splicing of endogenous *DMD* exon 39

**Figure 5.**
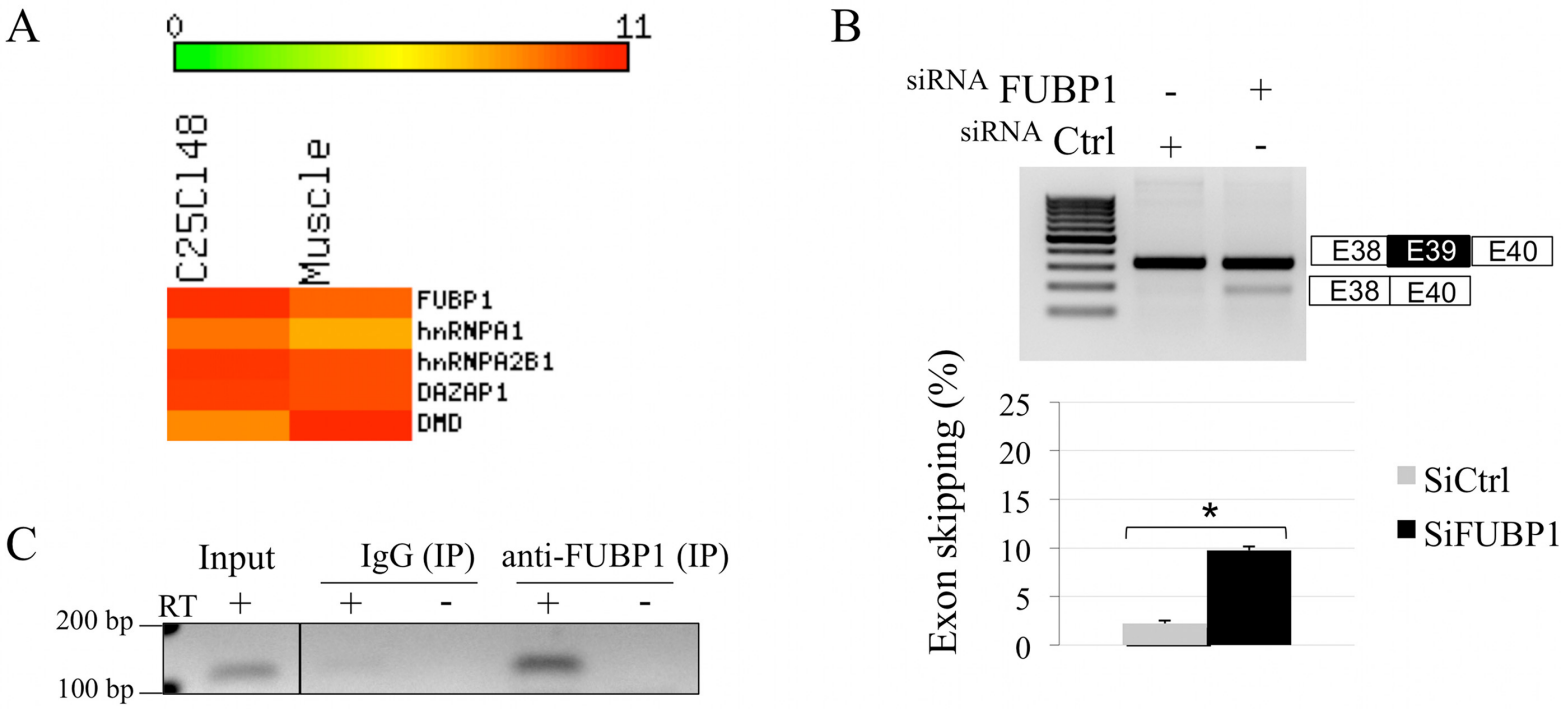
FUBP1 positively regulates the inclusion of endogenous *DMD* exon 39 and binds to the O region *in vivo*. (**A**) Heat map of Affymetrix whole transcriptome microarray data showing comparable expression levels of the selected splicing factors in C25cl48 cells and skeletal muscle tissue, including FUBP1. The color scale representing gene normalized intensity is shown above the Heat map. (**B**) Depletion of FUBP1 by siRNA induces skipping of endogenous *DMD* exon 39. C25cl48 myoblasts were transfected with siRNAs targeting either *FUBP1* (siFUBP1) or *firefly luciferase* (siCtrl). Upper panel: RT-PCR analysis of endogenous *DMD* transcripts using primers specific for *DMD* exons 38 (forward) and 40 (reverse) shows exon skipping of *DMD* exon 39 under FUBP1 depletion. Lower panel: exon skipping quantification and statistical analysis were as in Figure 2D. (**C**) Binding of endogenous FUBP1 to the *DMD* pre-mRNA in the O region by RNA-ChIP experiment. C25cl48 cell lysates were subjected to RNA-ChIP using an anti-FUBP1 (Genetex) antibody or normal mouse IgG as control. The *DMD* intron 38 containing region O (111bp) was amplified by RT-PCR from RNA corresponding to 0.5% of the input (input) and immunoprecipitated (IP) RNAs. Amplified fragments were loaded on a 2% agarose gel along with RT negative controls (–).

To assess the physiological significance of our findings, we checked the presence of the studied splicing factors in the C25cl48 myoblasts compared to skeletal muscle. Microarray gene expression data displayed high and comparable level of *FUBP1* transcript in human skeletal muscle and in the C25cl48 cells, confirming it represents a suitable cellular model (Figure 5A). The three other studied splicing factors (hnRNPA1, hnRNPA2B1 and DAZAP1) were also comparably expressed in muscle tissue and C25cl48 cells. We next examined the effect of FUBP1 depletion on the splicing of the endogenous *DMD* exon 39. The C25cl48 cells were treated with a siRNA targeting either *FUBP1* or *firefly luciferase* as a control and the splicing pattern of endogenous *DMD* exon 39 was analyzed (Figure 5B). FUBP1 depletion induced a statistically significant increase in the proportion of dystrophin transcripts lacking exon 39, showing that FUBP1 is required for proper recognition of *DMD* exon 39 during splicing in muscle cells. Finally, we checked the ability of endogenous FUBP1 to bind to the ISE element *in vivo* by performing an RNA-ChIP experiment using the C25cl48 myoblasts. As shown in Figure 5C, the RNA fraction immunoprecipitated with FUBP1 was clearly enriched in intron 38 sequences compared to the RNA fraction immunoprecipitated with the normal IgG. Altogether, these results indicate that, in the C25cl48 myoblasts, endogenous FUBP1 binds to the *DMD* pre-mRNA in the ISE-containing intron 38 region and that FUBP1 is required for correct splicing of exon 39 of the *DMD* gene.

## DISCUSSION

Here, we thoroughly investigated the molecular basis for partial in-frame exon skipping of *DMD* exon 39 harboring the c.5480T>A (p.Leu1417*) nonsense mutation in a patient with an attenuated phenotype. We showed that loss of exon identity is caused by the creation of an ESS that recruits splicing-repressor proteins. This study also yielded important insights into how normal splicing of *DMD* exon 39 is regulated. Notably, we uncovered that FUBP1, a single-stranded DNA- and RNA-binding protein, positively regulates *DMD* exon 39 splicing, and we identified an ISE in intron 38 that promotes exon recognition.

### ESS-mediated skipping of nonsense mutation-containing *DMD* exon 39

BMD-associated nonsense mutations are almost exclusively found in *DMD* exons 23–42, where mono- or multi-exon skipping maintains an open reading frame (8,9). While exon skipping events induced by exonic mutations are often attributed to loss of an ESE, the mutation can also promote exon skipping through gain of an ESS (30), or from the combination of both mechanisms (31). Our earlier experiments favored the ESS model and highlighted the role of newly created hnRNPA1-specific binding sites in the defective splicing of mutated *DMD* exon 31 (32). The c.5480T>A in exon 39 was also predicted *in silico* to create an hnRNPA1 binding site containing the core UAG sequence (33). Our experimental data showed the role of hnRNPA1, but also of hnRNPA2/B1 and DAZAP1 in the function of the splicing silencer sequence that is created by the mutation. While hnRNPA1 and the related hnRNPA2 protein are two well-known splicing-inhibitory factors (34), DAZAP1 is a more recently identified hnRNP protein implicated in several cellular processes, including transcription, mRNA translation and RNA splicing (35). Its role in splicing is not clearly established yet as only a few studies are available. DAZAP1 has been reported to promote splicing in exonic and intronic locations either directly by interacting with RNA elements in alternative exons or nearby introns, or indirectly through binding to members of hnRNPs, in particular by competing with and neutralizing splicing inhibitors in the hnRNP A1 family (36,37). A negative role of DAZAP1 on splicing was evidenced only in two previous studies in the context of pathological mutations involving *NF1* exon 37 and *BRCA1* exon 18 (18,26). Likewise the c.5480T>A mutation in the *DMD* gene, an exonic mutation created a new ESS element that was recognized by hnRNPA1/A2 as well as DAZAP1 and resulted in partial exon skipping. However contrary to what we observed with our *DMD* minigenes, the siRNA-mediated depletion of DAZAP1 alone in HeLa cells did not affect the splicing of the *NF1* or *BRCA1* mutant exon, and only when the three factors hnRNPA1, hnRNPA2 and DAZAP1 were concomitantly depleted, a synergistic effect leading to exon inclusion was seen.

In the *DMD* minigenes, the knockdown of DAZAP1 in C25cl48 myoblasts established for the first time the specific silencing effect of DAZAP1 on exon inclusion, which was also seen under overexpression conditions (Figure 2D and E). We considered that the restored exon 39 inclusion seen upon DAZAP1 depletion could not be attributed to the concomitant hnRNPA1 depletion because of the higher exon inclusion level (and in consequence lower exon skipping level) seen under si-DAZAP1 compared to si-hnRNPA1 in C25cl48 cells (40% and 48% exon skipping, respectively). The bound sequence in *DMD* mutated exon 39 (GAAUAG) is resembling but does not match perfectly any of the previously described DAZAP1 binding motifs in human genes (GGUUAG, GCUUAG, ACUUAG, GUAACG, AGAUAU, AAUUUA, AGUAGG) (18,26,37). Our data provides additional lines of evidence that DAZAP1 could negatively regulate exon inclusion. Whether DAZAP1 directly binds to the RNA sequence, which is also recognized by hnRNPA1 or acts through its interaction with hnRNPA1 and hnRNPA2/B1 remains to be elucidated.

The signaling network to specify splicing is particularly dense within and around exons (38) and the splicing outcome of the c.5480T>A mutation in *DMD* exon 39 is most likely a result of the cooperative as well as the antagonistic effects of multiple splicing factors. In this study, we could establish the role of one protein (FUBP1) that contributes to the recognition of endogenous *DMD* exon 39, thus partly counteracting the effect of the ESS.

### FUBP1 is required for proper splicing of endogenous *DMD* exon 39

FUBP1 was originally identified as a single stranded DNA-binding protein that modulates c-*Myc* mRNA levels by binding to FUSE, an A/T-rich element located 1.7 kb upstream of the *c-myc* promoter. FUBP1 also has functions in RNA metabolism as a member of the AU-rich element (ARE) binding protein family (translation or stabilization of several cellular and viral mRNA species) (review in (27)). Thus, by binding to DNA and RNA, FUBP1 modulates gene expression, regulates mRNA stability and functions as an ATP-dependent DNA helicase. FUBP1 involvement in splicing regulation was unanticipated until two recent studies demonstrated that FUBP1 inhibits splicing of *triadin* exon 10 through its binding to an ESS in a chimeric minigene context (19), or conversely promotes splicing in the context of the oncogene *MDM2* pre-mRNA (39). Still, the role of FUBP1 in splicing remains largely unknown. Here, we first showed that FUBP1 is a key positive splicing regulator of *DMD* exon 39 in a minigene context by means of functional studies (overexpression and siRNA experiments). Then, the siRNA-mediated depletion of FUBP1 in C25cl48 muscular cells also confirmed its physiological function for proper splicing of endogenous *DMD* exon 39.

### FUBP1 binds to an UG-rich intronic splicing enhancer upstream of *DMD* exon 39

Little is known about the sequence and characteristics of FUBP1 cognate RNA targets for splicing. In the two recently published studies, both the identified exonic (*triadin* exon 10) and intronic (*MDM2*) SREs are AU-rich sequences and display characteristics of binding sites for (ARE)-binding proteins in 3′UTR of transcripts. Here, a functional screening based on systematic deletion analysis and site-directed mutagenesis in the *DMD* exon 39 minigenes allowed us to delineate an intronic region located about 80 bp upstream of *DMD* exon 39 that acts as an ISE on exon 39 splicing. The identified ISE contains an UG-rich RNA sequence (site O) that is similar to the previously identified optimal DNA binding motif for the KH domains of FUBP1 (29). Specific interaction of FUBP1 with RNA probes containing the wild-type ISE element was independently confirmed with RNA pull down and RNA EMSA experiments. The selected mutations (O-mut and UGU-mut) strongly weakened FUBP1 binding (Figure 4D and E), stressing the role of the site O in the recruitment of the protein. In addition, we could evidence the direct recruitment *in vivo* of FUBP1 on *DMD* pre-mRNA in the ISE region (Figure 5C).

The FUBP family members (FUBP1, and the two homologs FUBP2 and FUBP3) contain four KH domains connected by a flexible Gly-rich linker (40). KH domains are reported to bind ssDNA and RNA with low micromolar affinity, but they are typically found in multiple copies in proteins associated with transcriptional and translational regulation, along with other cellular processes (41). The clustering of KH domains may increase nucleic acid recognition and specificity by augmenting recognition surface or by including neighboring structural motifs (41). It was shown that, together, the KH3 and KH4 domains of the K homology splicing regulator protein (KSRP or FUBP2) bind RNA ligand more tightly than each does separately (42). In our RNA pull down experiments (Supplementary Figure S2), we observed that the standard WT RNA probe that contains only two predicted binding sites did not bind FUBP1 whereas the extended (ug)6u5 RNA probes containing six additional potential sites did. In the FUSE ssDNA sequence, particular KH domain-binding tetrads are more crucial for FUBP1 binding, notably the two outer regions of strong interference that include the triplet TGTs, also present in all ssDNA SELEX consensus sequences (29). Similarly, we evidenced the importance of the UGU motif in RNA sequences bound by FUBP1. On two occasions, we could observe that the binding of FUBP1 was strongly decreased when the UGU motif at the 3′ extremity of the RNA probes used in RNA pull down and RNA EMSA experiments was mutated. This is illustrated in Supplementary Figure S2 (compare (ug)6u5-WT and (ug)6u5-5480A RNA probes, see Supplementary S3 for sequences), and Figures 4D and E (compare O-WT and UGU-mut RNA probes). Thus, our study has provided some clues about the specific features of the molecular recognition of RNA motifs by the KH domains of FUBP1. However, more research remains to be done to fully characterize the RNA motifs targeted by FUBP1, and to better understand how this protein participates in pre-mRNA splicing. A recent study showed that FUBP1 is involved in alternative splicing regulation in a position-dependent manner, and functions as a second-step repressor (19). Our data indicates that this protein may also be essential for inclusion of constitutively spliced exon.

In conclusion, this study illustrates how in-depth investigation of disease-causing mutations in human genes contribute to basic knowledge of fundamental cellular processes. We characterized the splicing factors that are recruited to the ESS created by the c.5480T>A mutation in exon 39, and repress its inclusion in the mature transcripts. Moreover, we identified a splicing factor required for efficient normal exon 39 inclusion, and we localized one new UG-rich ISE that can recruit this factor. This data further extends the repertoire of known ISEs, which are not as well characterized as the other SREs (36). It is also worth mentioning that this ISE is the first one identified in the *DMD* gene for inclusion of constitutive exons. A better knowledge of SREs in the *DMD* gene may prove useful in the context of the ongoing therapies for DMD targeting mRNA splicing such as the antisense-mediated exon skipping and the *trans*-splicing approach (15,43).

## SUPPLEMENTARY DATA

Supplementary Data are available at NAR Online.

SUPPLEMENTARY DATA
