# Supplementary material for: FUBP1: a new protagonist in splicing regulation of the DMD gene
Source: Nucleic Acids Res. 2015 Feb 6;43(4):2378–89. doi: 10.1093/nar/gkv086 (PMC4344520; doi:10.1093/nar/gkv086)
Supplement: SUPPLEMENTARY DATA [file supp_43_4_2378__index.html]

FUBP1: a new protagonist in splicing regulation of the DMD gene — FUBP1: a new protagonist in splicing regulation of the DMD gene — SUPPLEMENTARY DATA 

# FUBP1: a new protagonist in splicing regulation of the *DMD* gene

## SUPPLEMENTARY DATA

**Files in this Data Supplement:**

- SUPPLEMENTARY DATA
- SUPPLEMENTARY DATA
