# Supplementary material for: FUBP1: a new protagonist in splicing regulation of the DMD gene
Source: Nucleic Acids Res. 2015 Feb 6;43(4):2378–89. doi: 10.1093/nar/gkv086 (PMC4344520; doi:10.1093/nar/gkv086)
Supplement: SUPPLEMENTARY DATA [file supp_gkv086_nar-02601-a-2014-File008.pdf]

Supplementary Material

Protocol for sample preparation for mass spectrometry analysis

In-Gel Digestion

Gel bands were washed (1 mL of water and then 1 mL of 25 mM NH_4HCO_3), destained twice (1 mL of 50 % acetonitrile in 25 mM NH_4HCO_3), and then dehydrated twice (1 mL of 100 % CH_3CN). After reduction of disulfide bridges with 250 µl of 10 mM DTT at 56°C for 45 min, the supernatant was discarded and cysteins were alkylated with 250 µl of 55 mM iodoacetamide during 30 min under shaking in darkness. Gel bands were washed twice with 1 ml of 50 % acetonitrile in 25 mM NH_4HCO_3, then dehydrated with 1 mL of 100 % CH_3CN, and finally dried at room temperature. Ten microliters of a trypsin solution (Sequencing Grade Modified Trypsin, Promega, Madison, USA), at a concentration of 0.0125 µg/µL in 25 mM NH_4HCO_3, were added to each gel band that were kept on ice for 15 min. Sixteen microliters of 25 mM NH_4HCO_3 were added and the samples were kept another 15 min at room temperature. Digestion was performed overnight at 37°C. Peptides were extracted from the gel by additing 50 µl of 2 % formic acid. Samples were sonicated in an ultrasonic bath for 10 min and supernatants were recovered. Peptides were then extracted twice from gel by addition of 100 µL of a solution made of 80 % acetonitrile and 2 % formic acid. The supernatants were recovered, pooled and dried under vacuum. Peptides were then resuspended in 10 µl of 2 % formic acid before LC-MS/MS analysis.

LC-MS/MS of peptides

The protein digests were analyzed using a High Capacity ion trap mass spectrometer (Amazon; Bruker Daltonik GmbH, Bremen, Germany), interfaced with a nano-HPLC U3000 system (Thermo Scientific, Waltham, USA). After concentration on a pre-column (Thermo Scientific, C18 PepMap100, 300 μm × 5 mm, 5 μm, 100 A), the peptides were separated

with a reversed-phase capillary column (Thermo Scientific, C18 PepMap100, 75 μm × 250 mm, 3 μm, 100 A) at a flow rate of 0.3 μL/min using a gradient from 2 % to 40 % acetonitrile in 15 min, and eluted directly into the mass spectrometer. Proteins were identified by MS/MS by information-dependent acquisition of fragmentation spectra of multiple charged peptides. Up to three data-dependent MS/MS spectra were acquired in positive ion mode. MS/MS raw data were analyzed using Data Analysis software (Bruker Daltonik GmbH, Bremen, Germany) to generate the list of peptides masses. The NCBI non-redundant database (NCBInr, release 20120115) was queried locally using the Mascot search engine v.2.2.07 (Matrix Science, http://www.matrixscience.com) and with the following parameters: Homo Sapiens for the taxonomy, trypsin as enzyme, one allowed trypsin missed cleavage, carbamidomethylation of cystein as fixed modification, oxidation of methionine as variable modification. The mass tolerance was set to 0.6 Da in both MS and MS/MS mode. Proteins were validated provided that they were identified by at least with two peptides with a p value <0.05.
