# Supplementary figures and images for: FUBP1: a new protagonist in splicing regulation of the DMD gene
Source: Nucleic Acids Res. 2015 Feb 6;43(4):2378–89. doi: 10.1093/nar/gkv086 (PMC4344520; doi:10.1093/nar/gkv086)

**Supplementary Figures**


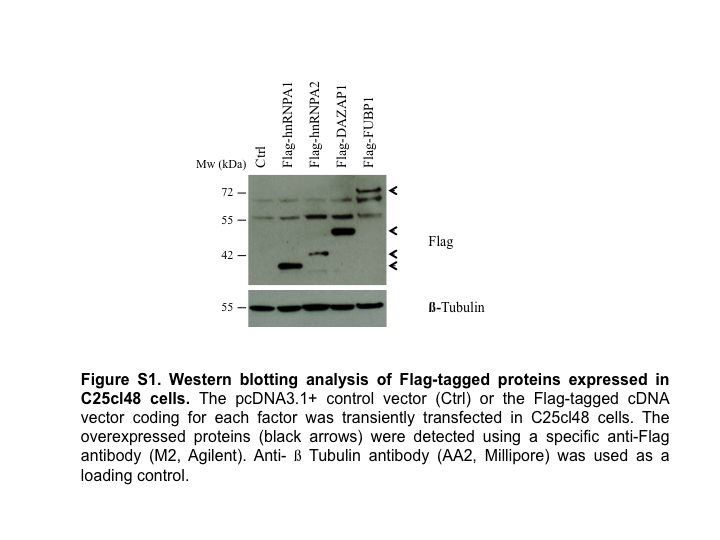


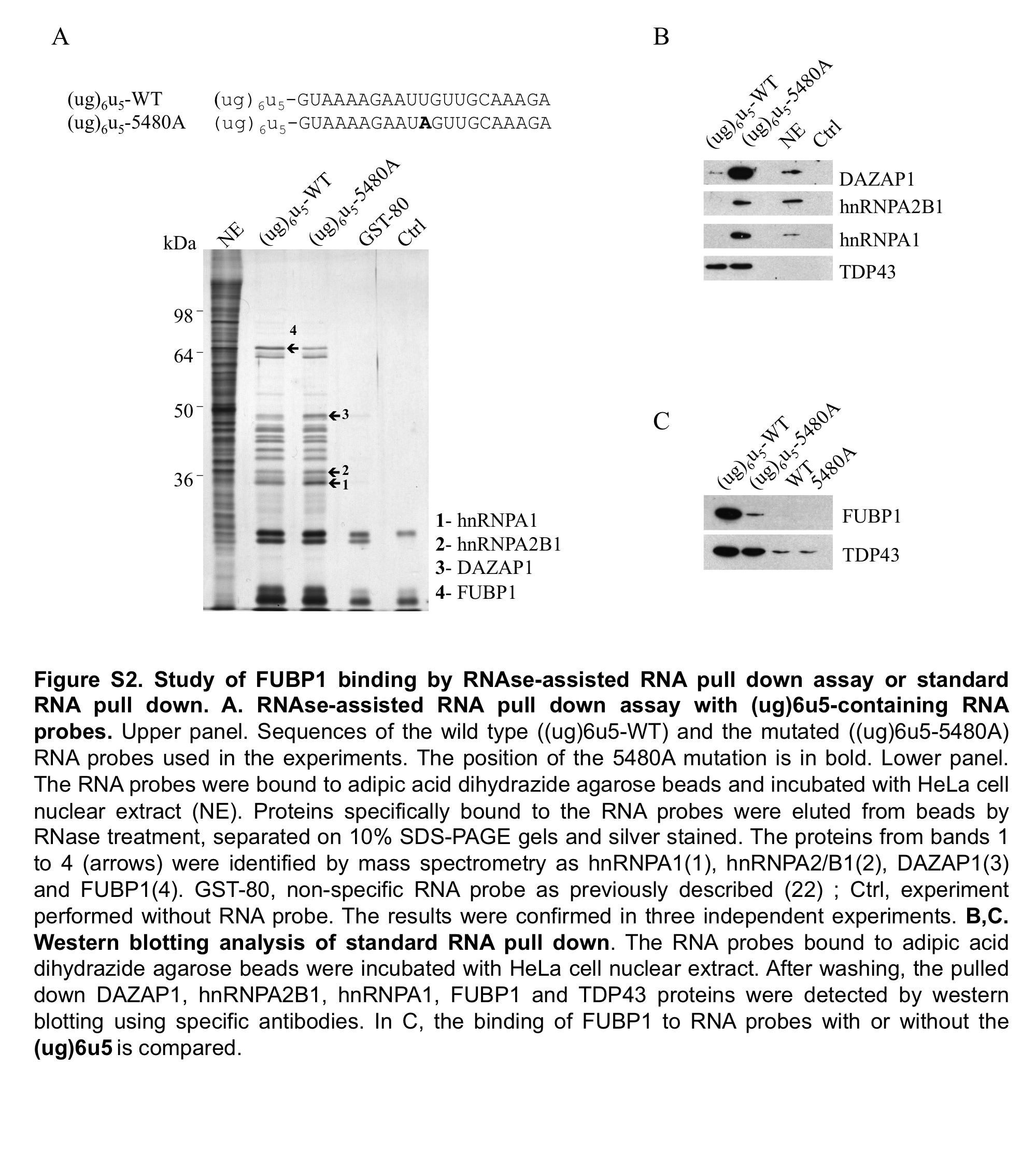


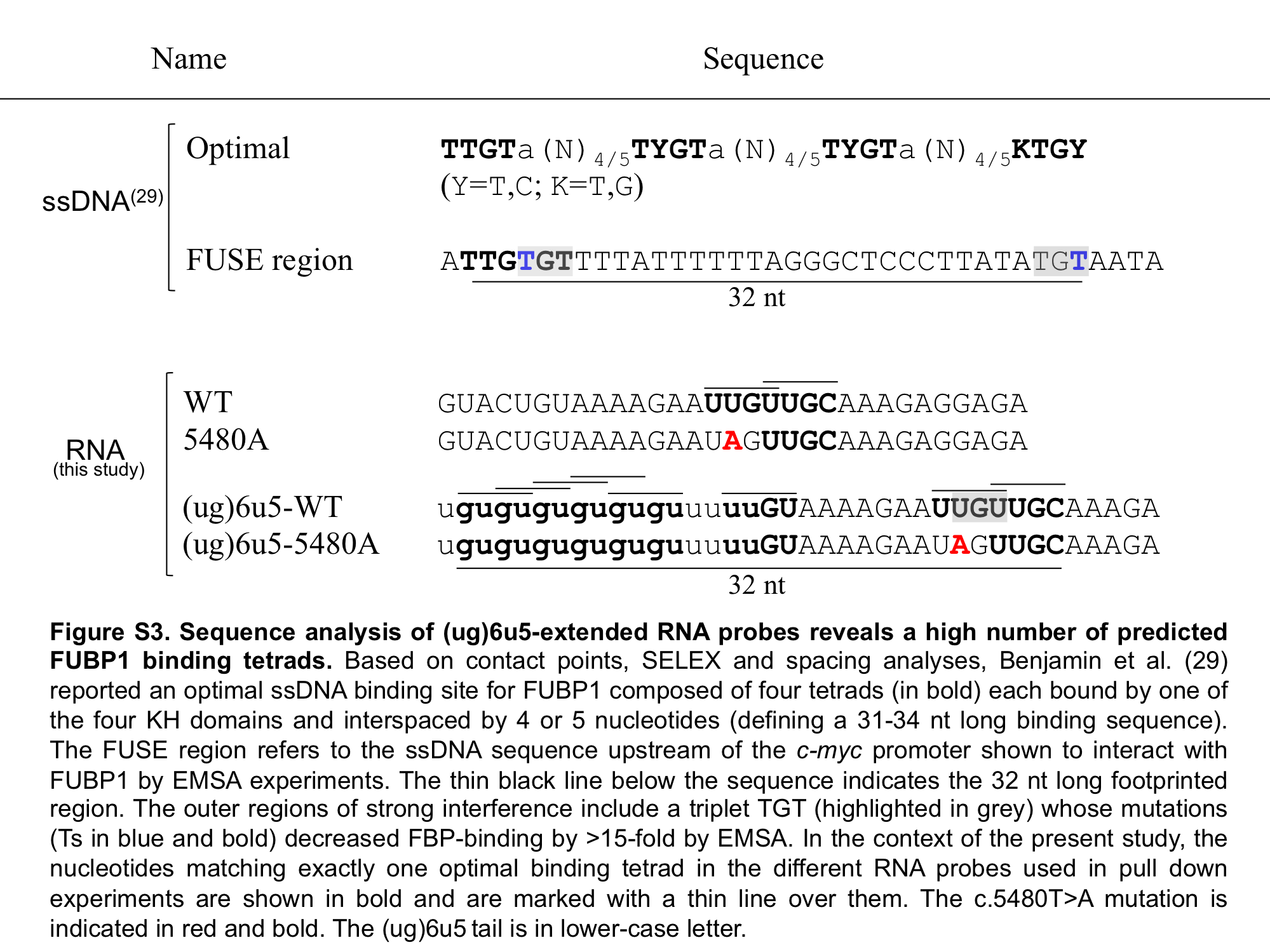


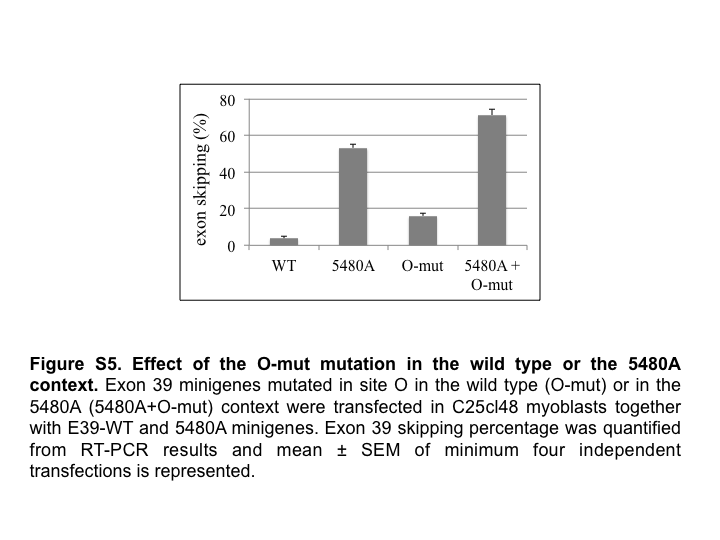

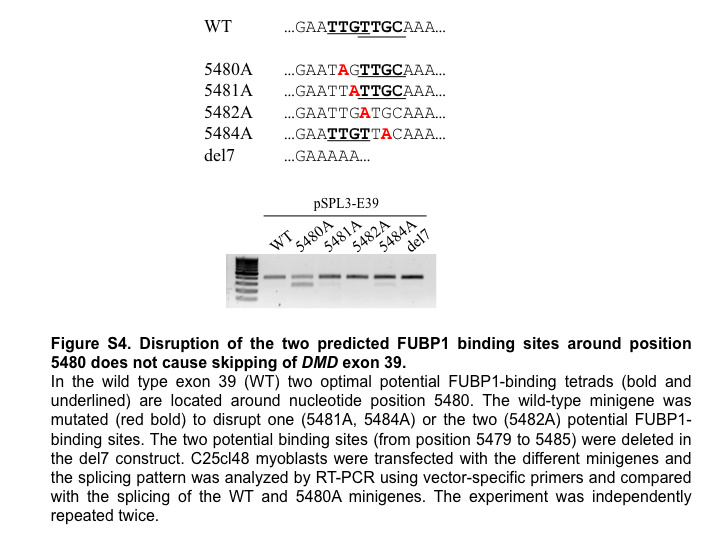


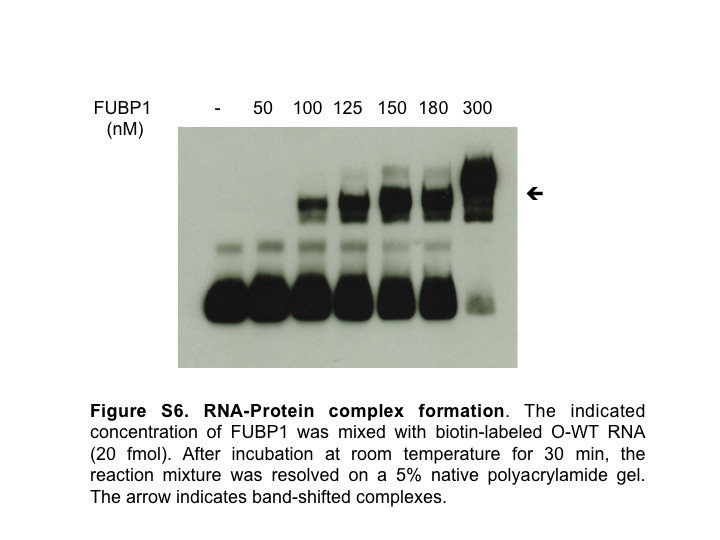

Supplement: SUPPLEMENTARY DATA [file supp_gkv086_nar-02601-a-2014-File007.docx]
